# Randomized blinded trial of standardized written patient information before total knee arthroplasty

**DOI:** 10.1371/journal.pone.0178358

**Published:** 2017-07-05

**Authors:** Benedicte Eschalier, Stephane Descamps, Bruno Pereira, Hélène Vaillant-Roussel, Guillaume Girard, Stephane Boisgard, Emmanuel Coudeyre

**Affiliations:** 1 Département de Médecine Générale, Faculté de Médecine, Université Clermont Auvergne Clermont-Ferrand, France; 2 Service de Chirurgie Orthopédique et Traumatologie, CHU Clermont-Ferrand, C-BIOSENSS, Université Clermont Auvergne, Clermont-Ferrand, France; 3 Direction de la Recherche Clinique et de l’Innovation, CHU Clermont-Ferrand, Clermont-Ferrand, France; 4 Service de Médecine Physique et de Réadaptation, CHU Clermont-Ferrand, France, INRA, Université Clermont Auvergne, Clermont-Ferrand, France; Harvard Medical School/BIDMC, UNITED STATES

## Abstract

**Background:**

The effect of patient education before total knee arthroplasty (TKA) is controversial. No consensus exists about the optimal content of educational interventions. In a previous study, we developed and validated an educational booklet on the peri-TKA management of knee osteoarthritis.

**Purposes:**

Our primary purpose was to evaluate the impact of the educational booklet on knowledge among patients awaiting TKA.

**Patients and methods:**

This randomized controlled single-blind trial evaluated standard information by the surgeon with or without delivery of the educational booklet 4–6 weeks before primary noncomplex TKA in patients aged 55–75 years with incapacitating knee osteoarthritis. Patients were enrolled at a French surgical center between June 2011 and January 2012. A patient knowledge score was determined at baseline, on the day before TKA, and 3–6 weeks after TKA, using a self-administered questionnaire developed for our previous study. The assessor was blinded to group assignment.

**Results:**

Of 44 eligible patients, 42 were randomized, 22 to the intervention and 20 to the control group, all of whom were included in the analysis. The groups were comparable at baseline. The intervention was associated with significantly better patient knowledge scores.

**Conclusions:**

An educational booklet improves knowledge among patients awaiting TKA. A study assessing the impact of the booklet combined with a exercise program would be helpful.

**Level of evidence:**

Level I, randomized controlled double-blind trial; see S1 CONSORT Checklist.

**Trial registration:**

clinicaltrials.gov #NCT01747759

## Introduction

Knee osteoarthritis results in loss of function due to muscle weakness, mobility and balance impairments, and cardiorespiratory deconditioning [1]. Knee osteoarthritis is the main reason for total knee arthroplasty (TKA), and the number of TKA procedures for knee osteoarthritis is expected to increase by 70% between 2005 and 2030 in the US [2]. TKA improves both function and pain. Pain and functional status before TKA predict the quality of the postoperative recovery [3].

Although recently published data support the delivery of patient education before TKA [4–8], the interventions studied vary widely and the results are somewhat conflicting. A 2011 meta-analysis of randomized controlled trials provided low-to-moderate evidence that a variety of preoperative interventions, including patient education, improved outcomes after TKA [5]. In a randomized trial, a booklet describing a combined exercise and educational intervention during the 4 weeks before TKA showed no significant differences versus standard care in terms of quality of life, pain, function, motion range, or muscle strength [6]. Nonsignificant decreases in hospital stay length and use of postoperative rehabilitation were noted in the intervention group. Another randomized controlled trial compared standard care to combined therapeutic education and functional rehabilitation involving two 30-minute one-on-one information sessions and two 90-minute group sessions on theoretical knowledge and execution of the exercises, without the delivery of written information [7]. The intervention was associated with improved function, decreased pain, increased analgesic consumption during the first 9 months, fewer primary-care physician visits, and diminished healthcare costs during the first 6 months. Finally, randomization to a single 40-minute preoperative educational session on rehabilitation combined with a booklet on the hospital stay and postoperative course was followed by decreases in surgical-ward stay length and costs compared to standard care but had no effect on function or pain [8]. In none of these trials were the theoretical and practical sessions supported by a validated and standardized printed document containing information on overall perioperative management in order to ensure uniformity of the information provided across healthcare providers, as suggested in current recommendations [9]. To our knowledge, no consensus has been developed regarding an information document similar to that described previously for low back pain but specifically designed for patients awaiting TKA [10]. In an open study, we used the method described by McClune et al. [11] to develop and validate an information booklet for patients awaiting TKA [12].

The primary purpose of our study was to evaluate the impact of our information booklet on TKA-related knowledge among patients scheduled for TKA. The secondary purposes were to measure the effect of the booklet on patient beliefs, surgery-ward stay length, discharge-to-home rate, and patient satisfaction.

## Patients and methods

### Study design

See S1 Protocol French version, S2 Protocol English version.

A single center, randomized controlled single blind two arm parallel group trial with a 1:1 allocation ratio evaluation standard information by the surgeon versus standard information by the surgeon plus delivery of a validated standardized information booklet with respect to 10 items questionnaire [12].

### Patient population

Patients were recruited at the Orthopaedic Surgery and Trauma Department of the teaching hospital in Clermont-Ferrand, France, among the total population of patients scheduled for TKA between June 2011 and December 2011. Patients follow up ended at the end of January 2012. Inclusion criteria were age 55 to 75 years, incapacitating knee osteoarthritis, scheduled TKA, ability to understand and cooperate with the study protocol, and informed consent to study participation. Exclusion criteria were institutionalization; cognitive impairments or behavioral disorders; difficulties with the French language that would preclude completion of the study assessments; previous TKA on the same knee; chronic inflammatory joint disease; and complex TKA.

Screening for eligibility and inclusion into the study were performed by the main study investigator. With the consent of the surgeon in charge of the patient, the main study investigator met with the patient immediately after the preanesthesia evaluation and suggested participation in the study. Patients who provided informed consent were randomized.

### Randomization procedure

A statistician who was not otherwise involved in the trial generated a randomization sequence with random block size using Stata software. The patients were told only that the study was designed to compare different preoperative management strategies. The outcome measures were assessed by the main investigator who was blinded to group assignment, of which only the surgeon was aware.

### Study intervention

The intervention consisted in having the outpatient clinic nurse hand the validated standardized information booklet to the patient at the end of the study inclusion visit just after the preanesthesia visit, i.e., 4–6 weeks before TKA. Patients were asked to read the booklet carefully several times. Patients in the intervention group and control group received the standard information delivered orally by the surgeon.

The following data were recorded for each patient: age, sex, body mass index, history of joint replacement surgery; social and occupational data; factors relevant to patient destination at discharge including patient wishes and the Risk Assessment and Prediction Tool (RAPT) score [13,14]; and function using the WOMAC scale [15,16].

Patient knowledge, beliefs, and satisfaction were evaluated using self-administered questionnaires delivered to the patient by the main study investigator. The questionnaires were completed at the end of the study inclusion visit (T0), at the hospital on the day before TKA (T1), and by telephone 3 to 6 weeks after TKA (T2). Patients who did not answer the telephone for the T2 evaluation received a reminder by land mail. Knowledge and beliefs were evaluated at all three time points. In addition, at T2 patient satisfaction with the information received was assessed, surgery-ward stay length was recorded, and whether the patient was discharged home or to a rehabilitation department was determined.

### Outcome measures

The primary outcome measure was patient knowledge about TKA assessed on the 10-item questionnaire used in an earlier study [12]. According to clinical relevance and previous works presented in literature, the primary endpoint was evaluated at T2 (last evaluation). Patient knowledge is widely used to assess educational interventions [17]. A correct answer to a question added 1 point to the score; incorrect answers and “don’t know” answers were scored 0; thus, the score could range from 0 to 10. The questions reflected the key messages delivered in the information booklet [12]. We are not aware of other tools designed to measure patient knowledge about TKA. The questionnaire is self-administered.

Secondary outcome measures included patient beliefs about topics addressed in the booklet, assessed using a 4-level Likert scale. According to sample size and clinical relevance, we decided to present two levels of responses: expected versus unexpected beliefs. We also recorded surgery-ward stay length, the proportion of patients discharged home and patient satisfaction with the information received about each of four items (hospital stay, surgery and surgical risks, available help in terms of money or home assistance, and ability to make changes to the home environment) assessed using a 4-level Likert scale. In addition, overall patient satisfaction with the information received was assessed on a numerical 0–10 scale.

### Statistical analysis

For type I and type II errors of 5% and 10%, respectively, 22 patients were needed per group to detect a difference of at least 2 points on the knowledge score, given that a previous pilot study [12] done by our group showed a standard deviation of 2. Because the two time-point evaluations were fixed according to usual follow-up, the risk of missing data (attrition bias) could be seen as minor. No interim analysis was planned and conducted.

Statistical analyses were performed using Stata software, version 12 (StataCorp, College Station, TX, USA). The tests were two-sided, with α = 0.05. Patient characteristics were described in each group as mean±SD or median (interquartile range, IQR) group for continuous variables, depending on distribution, and as the number of patients (%) for categorical variables. Groups were compared using the Chi-squared or Fisher exact test for categorical variables and Student t-test or Mann-Whitney test for quantitative variables. Assumption of normality (Gaussian distribution) was assessed using the Shapiro-Wilk test and homoscedasticity using the Fisher-Snedecor test. To study changes in knowledge scores over time, we built a mixed model taking into account within- and between-patient variability, considering the patient as a random-effect (slope and intercept) and to study randomisation group, time and their interaction as fixed effects considering an adjustment on physical activity. Mixed models were performed with an independent covariance structure which allows a distinct variance for each random effect within a random-effects equation and assumes that all covariances are zero. Results were expressed as regression coefficients (β), 95% confidence interval and Z statistic. Paired variables within groups were evaluated using the paired t-test or Wilcoxon test for quantitative parameters and the Stuart-Maxwell test for categorical variables.

### Ethics

This study was approved by our regional ethics committee, i.e., the *Comité de Protection des Personnes Sud Est I*, under # 2010–50 approval number on November 15th, 2010. Inclusion process started in june 2011. The Clinical trials registering was made on December 10th, 2012 which is an opportunity offered by CT web site to make a posteriori registering. This procedure is in accordance with French laws in clinical research on usual care. The authors confirm that all ongoing and related trials for this drug/intervention are registered.

There was no deviation to the validated protocol. Written consent was obtained for all patients included in the study.

## Results

### Baseline data S1 Data File

**Fig 1:**
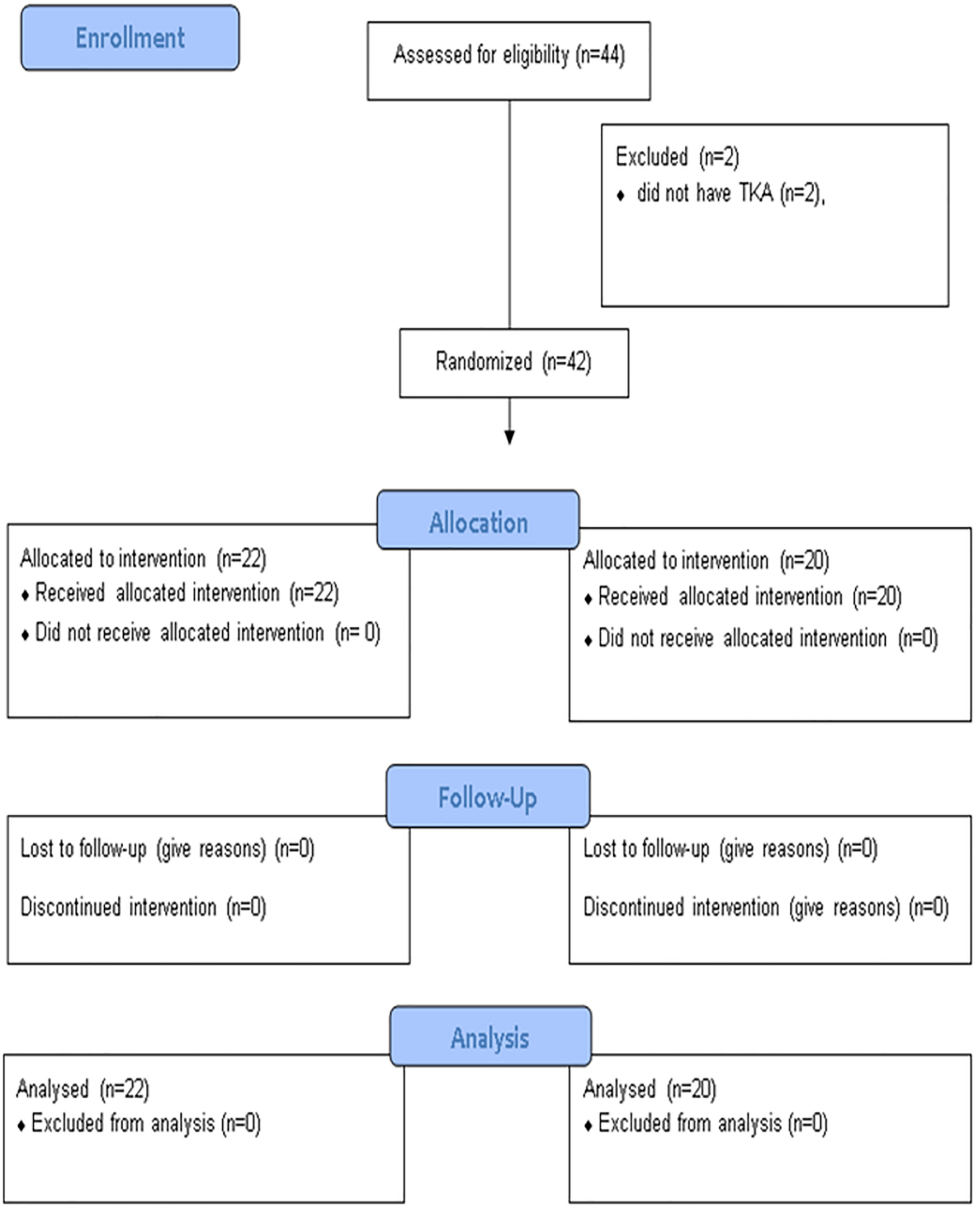
Study flow chart.

We included 44 patients between June 2011 and January 2012 (Fig 1), of whom 42 were randomized, 22 to the intervention and 20 to the control group; the remaining 2 patients had contraindications to anesthesia (Fig 1). All 42 patients completed the study according to the protocol and were analyzed in their group assigned by randomization (Table 1).

**Table 1:** Main baseline data in the two groups.

	Intervention Group	Control Group
	(n = 22)	(n = 20)
**Age (y), mean±SD**	68.1±4.7	66.8±5.8
**Females/Males, n**	10/12	10/10
**BMI (Kg/m**^**2**^**), mean±SD**	31.2±5.1	31.6±5.4
**Function (WOMAC score), mean±SD**	41.9±13.7	40.7±17.9
**Formal education** (Primary/Secondary/College), n	6/13/3	4/10/4
**Employed/retired, n**	2/20	3/16
**Preoperative physical activity, n (yes/no)**	9/12	14/6
**Frequency** <30 min x 3/week, ≥30 min x 3/week	7/2	4/9
**Type**		
Walking	3	3
Cycling	3	3
Swimming	1	2
Other	3	8
**Physiotherapy for knee OA, yes/no, n**	6/15	6/14
**History of TKA or THA, yes/no, n**	7/14	3/17
**RAPT score, mean±SD**	8.8±2.3	8.8±2.4
**Patient’s preferred location for postoperative rehabilitation (**home/ **r**ehabilitation unit)	19/3	12/8

BMI, body mass index; WOMAC, Western Ontario and McMaster osteoarthritis index OA, osteoarthritis; TKA, total knee arthroplasty; THA, total hip arthroplasty; RAPT, Risk Assessment and Predictor Tool for assessing the likelihood of discharge home

### Primary outcome measure

**Fig 2:**
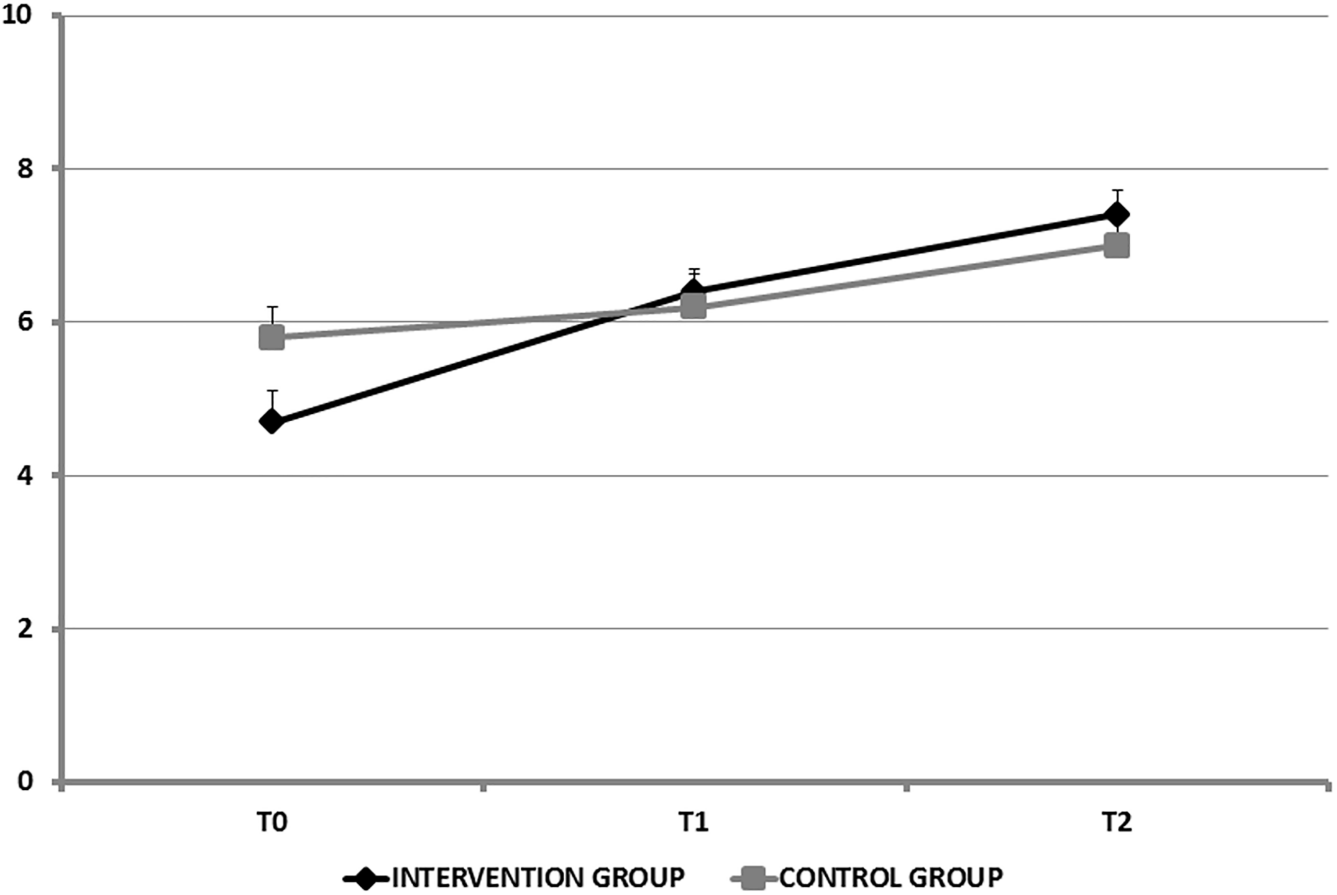
Changes in knowledge scores over time.

Mean total knowledge scores were not significantly different between the two groups at any of the three time points. Over time, knowledge improved in both groups and the improvement was larger in the intervention group (Table 2 and Fig 2). At baseline, the only individual item for which the proportion of correct answers differed significantly between the two groups was “Engaging in aerobic physical activities provide good preparation for TKA” this proportion was 55% in the control group and 22% in the intervention group (*P* = 0.03).

**Table 2:** TKA-related knowledge score values at the three study time points; The score range from 0 to 10, T0, study inclusion, 4–6 weeks before surgery; T1, day before surgery; T2, 3–6 weeks after surgery.

	Intervention Group (n = 22)	Control Group (n = 20)	P value
Knowledge score at T0, mean±SD	4.7±1.9	5.8±1.8	0.11
Knowledge score at T1, mean±SD	6.4±1.4	6.2±1.9	0.51
Knowledge score at T2, mean±SD	7.4±1.5	7±1.8	0.46

The random-effect model analysis showed a significant time-by-group interaction (*P* = 0.03, β = 0.70 [0.21; 1.20], Z = 2.12), which allow to explore results on intra-group analysis. From study inclusion (T0) to the day before TKA (T1), the knowledge-score improvement differed significantly (*P* = 0.015, β = 1.23 [0.24; 2.21], Z = 2.44) between the intervention and control groups (β = 1.69 [0.88; 2.49], Z = 4.10, *P* = 0.001 in the intervention group, β = 0.39 [-0.14; 0.92], Z = 1.44, *P* = 0.20, in the control group). From study inclusion (T0) to the last evaluation (T2), the mean improvement in knowledge was also significantly (*P* = 0.008, β = 1.40 [0.41; 2.38], Z = 2.78) greater in the intervention group (β = 2.68 [1.89; 3.47], Z = 6.63, versus β = 1.23 [0.69; 1.76], Z = 4.45 in the control group). From the day before TKA (T1) to the last evaluation (T2), the mean improvements were not significantly different (*P* = 0.70) between the two groups.

### Secondary outcome measures

The assessment of beliefs (Table 3) showed that from study inclusion (T0) to the day before TKA (T1), the proportion of expected responses in the intervention group was significantly greater for two items and significantly smaller for one item, compared to the control group. In the control group, the proportion of expected responses increased significantly for one item. From study inclusion (T0) to the last evaluation (T2), the proportion of expected responses increased significantly for three items in both groups.

**Table 3:** Changes in TKA beliefs over time.

	Intervention group			Control group		
	T0	T1	T2	T0	T1	T2
Physical activity is beneficial for your knee.	50,0	63,6	68,2	60,0	70,0	90,0*
Weight-bearing can be resumed less than 1 week after TKA.	45,5	54,5	81,8*	40,0	60,0*	90,0*
A rehabilitation unit stay will be required.	54,5	31,8*	45,5	45,0	40,0	35,0
TKA should not be performed until you are nearly unable to walk.	4,5	18,2	13,6	20,0	20,0	35,0
Strict bed rest is a treatment option for osteoarthritis.	63,6	59,1	81,8	70,0	75,0	85,0
TKA will completely eliminate your knee pain.	18,2	40,9*	36,4*	30,0	40,0	55,0
Assistive devices will be helpful.	54,5	63,6	63,6	35,0	55,0	60,0*
Preoperative physiotherapy sessions would have been helpful.	0,0	36,4*	27,3*	15,0	20,0	20,0

Data are percentage of expected beliefs; T0, study inclusion (4–6 weeks before surgery); T1, day before surgery; T2, 3–6 weeks after surgery

* Significant difference (p < 0.05) vs T0

Of the 42 patients, 31 (74%) were discharged home, 3/22 intervention-group patients and 7/20 (35%) controls. This difference was not significant. Surgery-ward stay length was not significantly different between the two groups (9.6 ±2.8 days and 8.8±2.0 days in the intervention and control groups, respectively).

Neither was there any significant between-group difference for overall patient satisfaction with the information received (7.6±1.5 in the intervention group and 7.7±1.7 in the control group) or for responses to individual items reflecting patient satisfaction with the four specific types of information (Table 4).

**Table 4:** Patient satisfaction with the information received.

	Intervention Group (n = 22)	Control Group (n = 20)	*P* value
**Information on the course of the hospital stay**			0.43
-highly satisfied	12	8	
-satisfied	4	8	
-partially satisfied	6	4	
-not at all satisfied	0	0	
**Information on TKA and TKA-related risks**			0.10
-highly satisfied	15	7	
-satisfied	5	8	
-partially satisfied	2	4	
-not at all satisfied	0	1	
**Information on possibilities of making changes to the home environment**			0.45
-highly satisfied	5	9	
-satisfied	7	5	
-partially satisfied	3	3	
-not at all satisfied	7	3	
**Information on available human and financial help**			0.76
-highly satisfied	4	2	
-satisfied	6	8	
-partially satisfied	6	4	
-not at all satisfied	6	6	

The data are the numbers of patients who gave each answer on the four-level Likert scale.

## Discussion

This study demonstrated that delivering written information to patients awaiting TKA improved their knowledge and, to a lesser extent, beliefs. The information booklet had no effect on surgical-ward stay length, proportion of patients discharged home without a stay in rehabilitation, or patient satisfaction with the information received.

The high prevalence of obesity among our patients, with a mean BMI of 31.1 Kg/m^2^, is consistent with previous data [17]. Obesity is a risk factor for both the development of knee osteoarthritis and greater progression of knee osteoarthritis. Many of our patients had insufficient physical activity, with 56% reporting some form of exercise but only 50% engaging in aerobic activity at the recommended frequency of at least 30 minutes 3 times a week [18].

The knowledge scores showed significantly greater increases over time versus baseline in the intervention group than in the control group. Absolute knowledge-score values were not significantly different between the two groups, despite the lower knowledge scores in the intervention group at study inclusion. The similarity of knowledge scores in the two groups at the last evaluation 3–6 weeks after TKA is probably ascribable to the experience acquired by the patients during the immediate postoperative period. When we looked at the knowledge scores for each individual item, we found that the greatest impact of the booklet was for the beneficial effects of regular physical activity before TKA.

Interestingly, at baseline, our patients had several erroneous beliefs. Of the 42 patients, 37 (88%) sought TKA only when they had become nearly unable to walk, although the quality of postoperative functional recovery depends in part on preoperative function [3]. Similarly, 39 (93%) patients believed that preoperative physiotherapy was unhelpful and 19 (45%) felt that engaging in physical activities might worsen their knee osteoarthritis [4,5]. Furthermore, 32 (76%) patients expected TKA to completely eliminate their knee pain, in contradiction with studies showing limited pain relief from TKA in some patients, particularly compared to total hip arthroplasty [19]. Higher patient expectations regarding outcomes are associated with lower postoperative patient satisfaction [20]. This fact strongly supports patient education before surgery, in order to improve the match between expectations and realistic outcomes. The information booklet had a limited effect in correcting these erroneous beliefs. In the intervention group, beliefs about the usefulness of preoperative physiotherapy and expectations regarding postoperative pain relief were improved. The booklet increased the proportion of patients who felt a stay in a rehabilitation ward was necessary. One possible explanation to this finding is that the booklet drew attention to the possibility of receiving postoperative rehabilitation, although the message in the booklet was that inpatient rehabilitation therapy was unnecessary. That the booklet failed to substantially change beliefs is unsurprising. Beliefs are deeply entrenched subjective convictions derived from personal experiences, life events, culture and feelings [21–23]. The discarding of erroneous beliefs requires a long and challenging psychological process driven by the repeated provision of information over time.

Overall patient satisfaction with the information received was high in both groups and was not improved by the booklet. The delivery of written documents to patients before surgery is routine, in particular for liability reasons. Patients may therefore experience information overload, which may prevent them from integrating relevant and appropriate messages. In addition, the high satisfaction in both groups diminished our ability to detect a significant effect of the booklet.

When we looked at the impact of written standardized information compared to the same information delivered orally by the study investigator, we noted that several intervention-group patients chose the “strongly disagree” response to the satisfaction questionnaire items about information on available financial and technical help and on the possibility of making changes to the home environment, and some of these patients added that this topic was not broached by the surgeon. Furthermore, a message delivered in a written document, even an evidence-based validated document developed by consensus, may not have as strong an impact as the same message delivered by the physician in charge of the patient [24–27]. Thus, written information completes but cannot replace oral information.

The booklet failed to decrease the length of stay in the surgical ward and the proportion of patients discharged to a rehabilitation ward. These results are unsurprising, as both variables are affected by multiple factors and are unlikely to be amenable to modification by simply delivering a booklet [28].

Our study has several limitations, particularly regarding the methodology used. The knowledge and beliefs questionnaires, although used in our earlier study [12], have not been scientifically validated. The patients may have misinterpreted some of the items, most notably in the beliefs questionnaire. Given the absence of relevant previously published data, we estimated our sample size based on the results of our pilot study and on our potential for patient recruitment. A clinical limitation is the nonstandardized nature of the information delivered orally by the various members of the surgical team. However, only the surgeon in charge of the patient was aware of group assignment.

Nevertheless, the significant changes in knowledge scores and in some of the beliefs demonstrate the usefulness of delivering standardized written information. Our findings support efforts to improve the education strategy for patients awaiting TKA. We are planning a complementary study aimed at evaluating the impact of combining oral information, the booklet, and a program of specific aerobic exercises. The information sessions will target knowledge gaps and erroneous beliefs identified during an educational evaluation.

## Conclusion

Delivering a standardized information booklet to patients 4–6 weeks before TKA significantly improved patient knowledge up to 6 weeks after the procedure same. At all time points, the impact of the booklet on patient beliefs was limited. The booklet did not significantly affect overall patient satisfaction with information, surgical-ward stay length, or the proportion of patients discharged to rehabilitation wards.

The booklet used in this study should prove useful as a foundation for building multidisciplinary education programs combining supervised exercises and standardized oral and written information in patients awaiting TKA.

## Supporting information

S1 CONSORT Checklist (DOC)

S1 Data File (XLSX)

S1 Protocol French version. (DOC)

S2 Protocol English version. (DOC)
