# Supplementary material for: Randomized blinded trial of standardized written patient information before total knee arthroplasty
Source: PLoS One. 2017 Jul 5;12(7):e0178358. doi: 10.1371/journal.pone.0178358 (PMC5497941; doi:10.1371/journal.pone.0178358)
Supplement: S1 Protocol — (DOC) [file pone.0178358.s003.doc]

**CENTRE HOSPITALIER UNIVERSITAIRE DE CLERMONT-FERRAND**

**PROTOCOLE D'ESSAI CLINIQUE**

Intitulé

**Evaluation d’une démarche d’éducation thérapeutique**

**pré-opératoire avant arthroplastie totale de genou.**

**Étude randomisée contrôlée**

**EPOP**

**Promoteur :** CHU de Clermont-Ferrand

**Investigateur principal :** Pr Stéphane Descamps

Service de chirurgie orthopédique et traumatologique

Hôpital Gabriel Montpied - CHU de Clermont-Ferrand

58 rue Montalembert

63003 Clermont-Ferrand cedex 1

**Lieux de l’étude :** Service de Chirurgie Orthopédique + Antenne de Médecine Physique et de Réadaptation

Hôpital Gabriel Montpied - CHU de Clermont-Ferrand

58 rue Montalembert

63003 Clermont-Ferrand cedex 1

**PLAN**

**Page**

**Résumé du protocole** **3**

**1- INFORMATIONS GENERALES 4**

**2- RATIONNEL DE L’ETUDE/JUSTIFICATION SCIENTIFIQUE 7**

2.1- Présentation du problème 7

2.2- Recommandations de la littérature 8

2.3- Intérêt de valider un outil d’information 8

**3- OBJECTIFS DE L’ETUDE 8**

3.1- Objectifs principaux 8

3.2- Objectifs secondaires 8

**4- PLAN EXPERIMENTAL 8**

4.1- Conception du livret 9

4.2- Etude contrôlée randomisée 10

4.2.1.- Plan expérimental et justification

4.2.2- Procédure de sélection

4.2.3- Randomisation

4.2.4- Critères d’évaluation

4.3- Réalisation pratique de l’étude 13

4.3.1- Déroulement pratique de l’étude pour les patients

4.3.2- Description des mesures prises pour réduire et éviter les biais

4.3.3 - Sortie prématurée de l’étude

**5**[**- CONSIDERATIONS STATISTIQUE**](#__RefHeading___Toc157336619)**S 15**

5.1- Justification du nombre de sujets à inclure 15

5.2- Analyses des données 15

**6- FAISABILITE DE L’ETUDE ET RESULTATS ATTENDUS 16**

6.1- Faisabilité de l’étude 17

6.2- Résultats attendus 17

[**7- ASPECTS LÉGAUX ET ÉTHIQUE**](#__RefHeading___Toc157336622)**S 18**

7.1- Conformité aux textes de références sur les recherches visant les soins courant 17

**7.2- Déclarations officielles 18**

[**7.3-**](#__RefHeading___Toc157336626) **Documentations de la Recherche 18**

7.4- Assurance qualité 18

7.5 Contrôle qualité 18

[**7.6-**](#__RefHeading___Toc157336627) **Information des sujets volontaires 19**

[**7.7-**](#__RefHeading___Toc157336628) [T](#__RefHeading___Toc157336629)**ranscription des données dans le cahier d’observation**s **19**

**7.8-** [**Confidentialité**](#__RefHeading___Toc157336630) **des données/Anonymat 19**

[**7.9-**](#__RefHeading___Toc157336631) **Amendements au protocole de la recherche 20**

7.10- Extension de la recherche 20

7.11- Archivage des données 20

[**7.12- Rapport final de la recherche**](#__RefHeading___Toc157336632) **21**

7.13- Budget de l’étude 21

[**7.14- Publications et propriétés des données**](#__RefHeading___Toc157336633) **21**

**8- REFERENCES 22**

RESUME

| **Titre** | Evaluation d’une démarche d’éducation thérapeutique pré-opératoire avant arthroplastie totale de genou. Étude randomisée contrôlée. |
| --- | --- |
| **Investigateur Coordonnateur** | Dr Stéphane Descamps |
| **Centre investigateur** | CHU Clermont-Ferrand |
| **Objectifs de l’étude** | **Objectif principal** :  Evaluer l’impact d’une démarche simple d’éducation thérapeutique sous la forme d’un livret d’information, grâce à des scores sur les connaissances et les croyances des patients gonarthrosiques en attente d’une prothèse totale du genou (PTG). |
| **Plan expérimental** | Etude, randomisée contrôlée en simple insu. |
| **Nombre de patients** | 22 patients dans le groupe intervention et 22 patients dans le groupe contrôle (informations et conseils habituellement prodigués par le chirurgien)  En l’absence de données publiées valides sur le critère principal de l’étude, le nombre de sujets par groupe est fixé à 22 patients. Avec un tel effectif, nous serions en mesure de montrer une différence significative de 2 points du score de connaissances coté de 0 à 10 avec un écart-type de 2 (supposé identique dans les deux groupes) pour un risque alpha de 5% et une puissance de 90%). |
| **Suivi par patient** | Entre 2 et 3 mois en fonction du délai entre la visite d’inclusion et l’intervention chirurgicale |
| **Durée prévisionnelle de l’étude** | 6 mois |
| **Déroulement de l’étude** | - V0 inclusion - Intervention (remise d’un livret d’information) - Visite pré-opératoire - Suivi téléphonique (+/- postal) à 6 semaines postopératoire |
| **Critères d’éligibilité** | **Critères d'inclusion** :   - Homme ou femme âgé(e) de 55 à 75 ans. - Patients souffrant d’une gonarthrose pour laquelle la mise en place d’une arthroplastie totale de genou est programmée par le chirurgien. - Patient ayant reçu l’information et acceptant de participer à l'étude. - Patient affilié à un régime de sécurité sociale (bénéficiaire ou ayant droit).   **Critères de non inclusion** :   - Patients institutionnalisés. - Patients ayant déjà reçu une arthroplastie de genou totale sur le genou homolatéral. - Patients souffrant de rhumatisme inflammatoire chronique. - Troubles cognitifs ou du comportement rendant impossible l’évaluation. - Troubles de la compréhension et d’expression de la langue française. - PTG sur genou complexe. |
| **Critères de jugement** | **Critère principal** :  L’objectif principal de cette étude est d’évaluer l’impact d’une démarche simple d’éducation thérapeutique sous la forme d’un livret d’information sur les connaissances et les croyances des patients gonarthrosiques en attente d’une prothèse totale du genou.  **Critères secondaires** :  Les objectifs secondaires sont la description de l’impact du livret sur la prise en charge du patient  (activité physique, douleur, durée d’hospitalisation en chirurgie, le taux de transfert en soins de suite et réadaptation et la durée du séjour) et la satisfaction vis-à-vis de l’information reçue. |

Mots Clés : gonarthrose, arthroplastie de genou, éducation thérapeutique, livret d’information, rééducation pré et post-opératoire.

**1- INFORMATIONS GENERALES**

**TITRE DE LA RECHERCHE** : Evaluation d’une démarche d’éducation thérapeutique pré-opératoire avant arthroplastie totale de genou. Étude randomisée contrôlée (EPOP).

**PROMOTEUR**

CHU de Clermont-Ferrand

**Délégation à la Recherche Clinique & à l’Innovation**

**Direction Générale Adjointe**

**Villa annexe IFSI**

**58 rue Montalembert**

**63003 Clermont-Ferrand Cedex 1**

Tél : 04.73.751.195 / Fax : 04.73.754.730

**INVESTIGATEUR PRINCIPAL**

Pr Stéphane Descamps

Service de Chirurgie Orthopédique et Traumatologique

Hôpital GABRIEL MONTPIED - CHU de Clermont-Ferrand

58 rue Montalembert

63003 Clermont-Ferrand cedex 1

**CO-INVESTIGATEURS**

Pr Stéphane Boisgard

Service de Chirurgie Orthopédique et Traumatologique

Hôpital Gabriel Montpied - CHU de Clermont-Ferrand

58 rue Montalembert

63003 Clermont-Ferrand cedex 1

Pr Emmanuel Coudeyre

Service de Médecine Physique et Réadaptation

CHU Clermont-Ferrand, Hôpital Nord

61 route de Chateaugay, BP 30056

63118 Cébazat

Dr Delphine Claus

Service de Médecine Physique et Réadaptation

CHU Clermont-Ferrand, Hôpital Nord

61 route de Chateaugay, BP 30056

63118 Cébazat

**PARTENAIRES ASSOCIES**

Bénédicte Eschalier (Interne en Médecine Générale)

Médecin en charge de l’étude

Service de Médecine Physique et Réadaptation

CHU Clermont-Ferrand Hôpital Nord

61 rue de Chateaugay, BP 30056

63118 Cébazat

Guillaume Girard (Interne)

Médecin évaluateur

Service de Chirurgie Orthopédique et Traumatologique

Hôpital Gabriel Montpied - CHU de Clermont-Ferrand

58 rue Montalembert

63003 Clermont-Ferrand cedex 1

Bruno Pereira, Docteur en Biostatistique

Direction de la Recherche Clinique

Hôpital Gabriel Montpied - CHU de Clermont-Ferrand

58 rue Montalembert

63003 Clermont-Ferrand cedex 1

**LIEUX DE L’ETUDE**

Service de chirurgie orthopédique + Antenne de Médecine Physique et Réadaptation

Hôpital Gabriel Montpied - CHU de Clermont-Ferrand

58 rue Montalembert

63003 Clermont-Ferrand cedex 1

**TRAITEMENT DES DONNEES**

Les données seront traitées par le service de chirurgie orthopédique du CHU de Clermont-Ferrand en collaboration avec la DRCI.

**CALENDRIER DE l’ETUDE**

Soumission au comité technique septembre 2010

Soumission CPP octobre 2010

Durée d’inclusion des patients : 6 mois (novembre 2010 à mai 2011)

Durée de participation à l’étude pour chaque patient : 1 h (inclusion) + 1 h (questionnaires de suivi)

Date de fin d’étude : juillet 2011

Analyse des données puis rapport de fin d’étude : 2012

# 2- RATIONNEL DE L’ETUDE/JUSTIFICATION SCIENTIFIQUE

## 2.1 Présentation du problème

## L’arthrose du genou conduit à des déficiences de force musculaire, de mobilité, d’équilibre et à un déconditionnement cardio-respiratoire qui contribuent à altérer les capacités fonctionnelles des patients et en particulier leur marche [1]. Elle est la principale cause de pose d’une prothèse totale de genou (PTG) avec aux Etats Unis une perspective d’augmentation de la pose de PTG pour gonarthrose de l’ordre de 70% en 2030. L’état fonctionnel des patients et le niveau de douleur sont régulièrement améliorés après mise en place d’une PTG et l’état physique et fonctionnel (score de WOMAC) pré-PTG est prédictif de la récupération dans les suites d’une arthroplastie [2]. Une rééducation menée en pré-opératoire pourrait permettre de mieux préparer les patients à l’intervention, améliorer le résultat fonctionnel et accélérer l’autonomie en post-opératoire et ainsi diminuer la durée de séjour et faciliter les modalités de retour au domicile (direct ou après séjour en établissement de MPR) [3].

2.2 Recommandations de la littérature

La Société Française de Médecine Physique et Réadaptation (SOFMER), reprenant les données de la littérature, associées au recueil des pratiques professionnelles recommande la réalisation d’une rééducation pré-opératoire avant arthroplastie totale de genou [3]. Cette rééducation doit comporter au minimum de la kinésithérapie associée à une éducation ciblée. Une prise en charge pluridisciplinaire comportant au minimum de l’ergothérapie et une éducation est souhaitable en particulier chez les patients les plus fragiles du fait de capacités fonctionnelles altérées, de comorbidités ou de problèmes sociaux. En revanche, la réalisation d’une kinésithérapie isolée n’est pas recommandée.

Les recommandations de la HAS [4] précisent le contenu de la kinésithérapie pré-opératoire qui doit associer de l’éducation thérapeutique (utilisation des aides techniques, transferts, escalier, déroulement de la phase post-opératoire) au travail analytique et à la kinésithérapie respiratoire.

L’ensemble des études analysées pour l’élaboration de ces recommandations font appel à des interventions éducatives mais la nature des informations délivrées est mal précisée et non standardisée.

2.3 Intérêt de valider un outil d’information

Un support papier est un instrument idéal pour diffuser des informations consensuelles et contribuer ainsi à l’éducation thérapeutique des patients [5]. Le but de l’éducation des patients est de modifier leur attitude et leur comportement vis-à-vis d’un problème de santé ce qui doit passer par une amélioration de leurs connaissances et de leurs croyances pouvant concourir à modifier leur comportement. Ce type de critère est largement utilisé dans les études d’impact de démarches éducatives [6].

Cependant, à notre connaissance, il n’existe aucun document d’information validé disponible pour les patients français avant arthroplastie totale du genou. Il est nécessaire de pouvoir éduquer les patients avant PTG afin de les préparer à l’intervention et faciliter leur retour rapide à domicile ainsi que la reprise de leurs activités quotidiennes. Cette éducation doit en premier lieu pouvoir s’appuyer sur un livret d’information dont le contenu issu de la médecine fondée sur les preuves soit validé à la fois par des experts mais également auprès de patients.

Nous avons validé un livret d’éducation élaboré conformément à la méthodologie proposée par Mc Clune [7] et aux recommandations de la Haute Autorité de Santé [8] sur la rédaction des documents d’information destinés aux patients. Ce livret a fait l’objet d’une publication [9].

**3- OBJECTIFS DE L’ETUDE**

L’objectif principal de cette étude est d’évaluer l’impact d’une démarche simple d’éducation thérapeutique sous la forme d’un livret d’information, grâce à des sores de connaissances et de croyances des patients gonarthrosiques en attente d’une prothèse totale du genou.

Les objectifs secondaires sont la description de l’impact du livret sur la prise en charge (activité physique, douleur, durée d’hospitalisation en chirurgie, le taux de transfert en soins de suite et réadaptation et la durée du séjour) et la satisfaction vis-à-vis de l’information reçue.

**4- PLAN EXPERIMENTAL**

Etude monocentrique contrôlée randomisée : une prise en charge usuelle prodiguée par le chirurgien associée à une prise en charge éducative simple sous la forme d’un livret d’information.

4.1 Intervention

Remise d’un livret d’information élaboré selon la a méthodologie proposée par Mc Clune [7] et conformément aux recommandations de la Haute Autorité de Santé [8] et ayant fait l’objet d’une publication [9].

4.2 Etude contrôlée randomisée

4.2.1 Plan expérimental et justification

Nous allons mettre en œuvre un essai contrôlé randomisé monocentrique comparant :

1. une prise en charge usuelle prodiguée par le chirurgien
2. une prise en charge usuelle prodiguée par le chirurgien associée à une prise en charge éducative simple sous la forme d’un livret d’information

L’essai contrôlé randomisé est la méthode de référence pour l’évaluation thérapeutique. Les patients randomisés dans le groupe contrôle n’auront pas de prise en charge spécifique en dehors de la prise en charge classique proposée par leur chirurgien. Ce choix permet de se placer dans la situation la plus représentative de la pratique clinique courante et il tient compte de l’absence de traitement de référence ayant démontré son efficacité.

Ce choix pose un certain nombre de problèmes méthodologiques car l’insu des patients, des chirurgiens, de l’équipe médicale et paramédicale responsable de la prise en charge des patients, ne sera pas possible. Par conséquent, il y aura un risque de biais de performance et d’évaluation. Pour tenir compte de ces risques, nous allons organiser une évaluation indépendante réalisée par un médecin non impliqué dans la prise en charge des patients (médecin évaluateur).

Les patients seront en insu des hypothèses, c'est-à-dire qu’ils seront informés que l’objectif de cette étude sera de comparer différentes prises en charge des patients en pré-opératoire. En revanche, le contenu de chaque prise en charge ainsi que les hypothèses initiales de l’étude ne leur seront pas détaillés. Cette stratégie devrait limiter les biais, en particulier le risque de biais d’évaluation. Cette méthode est acceptable éthiquement car les patients seront informés que pour des raisons scientifiques on ne peut pas leur expliquer toutes les hypothèses de l’étude. Les patients seront tous informés à la fin de l’étude des hypothèses et des résultats de l’étude s’ils le souhaitent.

4.2.2 Procédure de sélection

Les patients seront recrutés dans le service de chirurgie orthopédique du CHU de Clermont-Ferrand au sein de la cohorte de patients dont l’intervention pour PTG est programmée. Les patients devront répondre aux critères d’inclusion et de non inclusion décrits ci-dessous pour être inclus dans l’étude.

*Critères d'inclusion*

Les patients inclus dans cette étude devront répondre aux critères suivants :

- Homme ou femme âgé(e) de 55 à 75 ans.

- Souffrant d’une gonarthrose invalidante, résistante aux traitements pharmacologiques pour laquelle la mise en place d’une prothèse totale de genou est programmée par le chirurgien.

- Coopération et compréhension permettant de se conformer de façon stricte aux conditions prévues par le protocole.

- Acceptation de participer à l’étude (non opposition).

- Affiliation au régime de la sécurité sociale.

*Critères de non inclusion*

- Patients institutionnalisés.

- Patients ayant déjà reçu une arthroplastie totale de genou sur le genou homolatéral.

- Patients souffrant de rhumatisme inflammatoire chronique.

- Patients présentant des troubles cognitifs ou du comportement rendant impossible l’évaluation.

- Patients présentant des troubles de la compréhension et d’expression de la langue française.

- PTG sur genou complexe.

## 4.2.3 Critères d’évaluation

a)- Critère d’évaluation principal

Le critère principal d’évaluation sera un score de connaissances sur l’arthroplastie totale du genou élaboré spécifiquement pour l’étude ainsi que les croyances du patient (degré d’accord avec les informations contenues dans le livret évalué par une échelle de Likert à 4 niveaux). Ce critère sera évalué lors de la visite d’inclusion, la veille de l’intervention et à 6 semaines post-opératoires.

b)- Critères d’évaluation secondaires

Les autres critères d’évaluation sont l’impact du livret sur la prise en charge (activité physique, douleur, durée d’hospitalisation en chirurgie, taux de transfert en soins de suite et réadaptation, durée du séjour) et la satisfaction vis-à-vis de l’information reçue à 6 semaines post-opératoires.

## 4.3 Réalisation pratique du protocole

4.3.1 Déroulement pratique de l’étude pour les patients

***Visite de sélection*** *(réalisée lors de la consultation de programmation de l’intervention)*

Compte tenu du manque de disponibilité des cliniciens et en particulier des chirurgiens, l’inclusion des patients dans les essais clinique est difficile. Afin de palier cette difficulté, le screening et l’inclusion des patients seront réalisés par le médecin en charge de l’étude. Tous les patients pour lesquels la mise en place d’une prothèse de genou est programmée seront identifiés. Avec l’accord du chirurgien en charge du patient, le médecin en charge de l’étude rencontrera le patient à l’issue de la consultation de programmation de l’intervention, vérifiera les critères d’éligibilité et proposera au patient de participer à l’étude.

Si le patient est intéressé par cette étude, et après un délai de réflexion d’environ un mois (laps de temps entre la consultation chirurgicale de programmation de l’intervention et la consultation pré-opératoire d’anesthésie), une nouvelle visite sera organisée au moment de la consultation d’anesthésie. Au cours de cette consultation, les critères d’éligibilité seront vérifiés, le patient sera informé, et après obtention de son accord (non opposition) il sera randomisé.

Les données suivantes seront collectées dans le cahier d’observation :

- Données démographiques (âge, sexe)
- Données socio-professionnelles (milieu socio-économique, profession et statut professionnel (en activité, en arrêt de travail, en invalidité))
- Indice fonctionnel : échelle de WOMAC

***Evaluations****:*

Les évaluations seront réalisées à l’aide de questionnaires à l’issue de la visite d’inclusion, la veille de l’intervention et à 6 semaines post-opératoire par un entretien téléphonique avec relance par voie postale si nécessaire. Le médecin en charge de l’étude se chargera de noter les initiales et le numéro patient sur le CRF avant l’éventuel envoie ; il se chargera également de réceptionner et centraliser les auto-questionnaires postaux. Afin de limiter les biais, en particulier d’interprétation et de recueil des données, ces visites d’évaluation seront réalisées par le médecin évaluateur.

## 4.3.2 Description des mesures prises pour réduire et éviter les biais

Randomisation :

Compte tenu de l’absence d’insu dans cette étude, une randomisation par sujet sera réalisée. Une liste de randomisation par strates sera établie en fonction de l’âge, du sexe et des antécédents de PTG. La numérotation se fera par ordre chronologique des inclusions. La randomisation sera réalisée après l’inclusion via le dossier médical informatisé géré sous I2000 (logiciel gestion des patients du CHU de Clermont-Ferrand).

Simple aveugle :

Cette étude sera réalisée en simple aveugle en ce qui concerne l’intervention, le patient recevant un livret d’information ne pouvant l’ignorer. Le médecin en charge de l’évaluation ne connaitra pas le groupe dans lequel le patient a été inclus.

Le biais de contamination sera négligeable, la randomisation ayant lieu au minimum un mois avant la chirurgie, à l’issue de la consultation d’anesthésie. La remise du livret s’effectuant dans les suites immédiates. De plus l’évaluation des patients sera réalisée lors de leur admission dans le service limitant l’interaction entre patients et avec le personnel para-médical du service de chirurgie.

## 4.3.4 Refus et Sortie prématurée de l’étude

Tout patient aura la liberté de refuser de participer à l’étude sans conséquence pour sa prise en charge, en particulier per et post-opératoire.

Un patient sortira prématurément de l’étude dans les cas suivants:

- La personne décide de ne plus participer à l’étude.
- La personne est non compliante pour réaliser les évaluations prévues dans le protocole.

Pour toute sortie prématurée de l’étude, la date et la raison seront indiquées dans le cahier d’observation du sujet. Chaque sujet sortant prématurément de l’étude devra être remplacé par l’inclusion d’une nouvelle personne.

# 5- CONSIDERATIONS STATISTIQUES

5.1 Justification du nombre de sujets à inclure

En l’absence de données publiées valides sur le critère principal de l’étude, le nombre de sujets par groupe est fixé à 22 patients; en accord avec les capacités de recrutement du CHU de Clermont-Ferrand. Précisons néanmoins qu'avec un tel effectif, nous serions en mesure de montrer une différence significative de 2 points du score de connaissances de 0 à 10 avec un écart-type de 2 (supposé identique dans les deux groupes) pour un risque alpha de 5% et une puissance de 90%. En effet, le nombre de sujets nécessaire requis sous ces hypothèses est de 22 sujets par groupe.

5.2 Analyses des données

L’analyse statistique sera réalisée au niveau du service d’orthopédie du CHU de Clermont-Ferrand à l’aide du logiciel SPSS en collaboration avec Mr Bruno Pereira, biostatisticien de la DRCI du CHU de Clermont-Ferrand. L’analyse des données sera faite en intention de traiter c'est-à-dire que tous les patients randomisés seront analysés et chaque patient sera analysé dans le groupe dans lequel il a été randomisé, quelque soit le type d’information reçue. Elle sera conduite après recueil de toutes les données selon les méthodes usuelles. Ainsi, les variables qualitatives seront décrites en termes d’effectifs et de proportions. Les variables quantitatives seront décrites en termes d’effectifs, moyenne et écart-type associé, médiane, étendue et étendue interquartile. La comparaison entre les deux groupes utilisera le test de Student ou de Wilcoxon si l'hypothèse de normalité du score n'est pas vérifiée. Les comparaisons des critères qualitatifs (par exemple critère de jugement catégorisé) utiliseront le test du Chi-Deux ou de Fisher exact (si conditions de validité non respectées). Une régression linéaire, avec pour variable dépendante le score de connaissances, sera mise en œuvre afin d'investiguer les paramètres qui peuvent avoir une influence sur ce score. Le seuil de significativité des différents tests est fixé à 5%.

**6- FAISABILITE DE L’ETUDE ET RESULTATS ATTENDUS**

6.1 Faisabilité de l’étude

Les investigateurs impliqués dans le projet ont une grande expertise dans la prise en charge des malades atteints de gonarthroses, et sont compétents dans l’approche éducative des patients en attente d’arthroplastie totale du genou.

Le nombre de sujets a été fixé à 22 sujets par groupe. Ce recrutement se fera parmi une cohorte de plus de 200 patients opérés par an au CHU de Clermont-Ferrand.

6.2 Résultats attendus

Les résultats attendus sont une amélioration significative des connaissances des patients sur la prise en charge péri-chirurgicale de la PTG contribuant à une réduction de leurs croyances et une amélioration de leur satisfaction vis-à-vis de l’information reçue.

**7- ASPECTS LÉGAUX ET ÉTHIQUE**

## 7.1 Conformité aux textes de référence sur les recherches visant à évaluer les soins courants

Les techniques et les méthodes utilisées au cours de cette recherche étant habituellement réalisées, elle peut rentrer dans le cadre des **recherches visant à évaluer les soins courants** tels que définis par la loi n°2004-806 du 9 août 2004 (article L1121-1, 2° alinéa et article R1121-3 du code de la santé publique).

## 7.2 Déclarations officielles

## Comité de Protection des Personnes

Le protocole, le document d'information ainsi que le cahier d’observation de l'étude seront soumis pour avis au Comité de Protection des Personnes Sud Est I. La notification de l'avis favorable du CPP sera transmise au promoteur de l'étude.

**CCTIRS et CNIL**

Le protocole de l'étude et les questionnaires seront soumis à l'avis du Comité Consultatif sur le Traitement de l’Information en matière de Recherche dans le domaine de la Santé (CCTIRS) et à l’autorisation de la Comission Nationale Informatique et Libertés (CNIL). Le fichier informatique restera strictement confidentiel. En application de la loi « Informatique et Libertés », les patients pourront à tout moment exercer un droit d’accès, de rectification, et d’opposition à la transmission des données informatisées les concernant auprès des responsables de l’étude ; ce droit pouvant être exercé directement ou par l’intermédiaire du médecin de leur choix pour les données médicales les concernant, en application de la loi du 4 mars 2002 relative aux droits des malades et à la qualité du système de santé.

Aucune rémunération ne sera allouée aux volontaires.

L’investigateur s’engagera également à travailler en accord avec la Déclaration d'Helsinki de l’Association Médicale Mondiale (Tokyo 2004, révisée).

## 7.3 Documentations de l’étude

Avant de démarrer la recherche, l'investigateur fournira au représentant du promoteur

de la recherche une copie de son curriculum vitæ personnel daté et signé et comportant son numéro d’inscription à l’ordre des médecins.

La version du protocole avec ses annexes sera signée conjointement par l’investigateur et le représentant du promoteur. Le cas échéant, le responsable scientifique sera également signataire.

Lors de chaque nouvelle version du protocole, rendue nécessaire par des amendements et/ou demandes des autorités, un nouveau numéro et la date seront attribués et les mêmes signatures recueillies.

Chaque investigateur s'engagera à respecter les obligations de la loi et à mener la recherche selon les bonnes pratiques cliniques (B.P.C).

## 7.4 Assurance de Qualité

Un Attaché de Recherche Clinique (ARC) mandaté par le promoteur s’assurera de la

bonne réalisation de l’étude, du recueil des données générées par écrit, de leur documentation, enregistrement et rapport, en accord avec les Procédures Opératoires Standards mises en application au sein du CHU de Clermont-Ferrand et conformément aux Bonnes Pratiques Cliniques ainsi qu’aux dispositions législatives et réglementaires en vigueur.

## 7.5 Contrôle de Qualité

L’investigateur se porte garant de l’authenticité des données recueillies dans le cadre de l’étude et accepte les dispositions légales autorisant le promoteur de l’étude à mettre en place un contrôle de qualité.

L'investigateur coordinateur et les investigateurs associés acceptent donc de se rendre disponibles lors des visites de Contrôle de Qualité effectuées à intervalles réguliers par l’Attaché de Recherche Clinique. Lors de ces visites, les éléments suivant seront revus :

. Le document d’information patient et la signature des patients

. Respect du protocole de l'étude et des procédures qui y sont définies

. Qualité des données recueillies dans le cahier d'observation : exactitude, données manquantes, cohérence des données avec les documents "source" (dossiers médicaux, carnets de rendez-vous, originaux des résultats de laboratoire, etc.…)

. Gestion des produits éventuels.

## 7.6 Information des patients

Les patients ne pourront participer à cette étude qu’après avoir reçu une information de la part du médecin sur : le but de cette étude, la durée de leur participation, les procédures qui seront suivies.

L’ensemble de ces informations sera résumé sur un document d’information remis à chaque patient.

Ils pourront à tout moment exercer un droit d’accès, de rectification et d’opposition à la transmission des données informatisées les concernant auprès des responsables de l’étude ; ce droit pouvant être exercé directement ou par l’intermédiaire du médecin de leur choix pour les données médicales les concernant, en application de la loi du 4 mars 2002 relative aux droits des malades et à la qualité du système de santé.

##

## 7.7 Transcription des données dans le cahier d’observation

Toutes les informations requises par le protocole doivent être fournies dans le cahier d’observation et une explication donnée par l’investigateur pour chaque donnée manquante.

Les données devront être transférées dans les cahiers d'observation au fur et à mesure qu'elles sont obtenues. Les données devront être copiées de façon nette et lisible à l'encre noire dans ces cahiers (ceci afin de faciliter la duplication et la saisie informatique).

Les données erronées dépistées sur les cahiers d'observation seront clairement barrées et les nouvelles données seront copiées sur le cahier avec les initiales et la date par le membre de l'équipe de l'investigateur qui aura fait la correction.

## 7.8 Confidentialité des données

La confidentialité des données sera assurée par l’utilisation de la mention les trois premières lettres du nom et les trois premières lettres du prénom du patient sur tous les documents nécessaires à la recherche associé à un numéro patient (donné par ordre d’inclusion), ou par effacement par les moyens appropriés (blanc correcteur…) des données nominatives sur les copies des documents source, destinés à la documentation de la recherche.

Une liste de correspondance entre les éléments nominatifs et le numéro patient sera établie et conservée par le médecin en charge de l’étude. Elle sera détruite à la fin de l’étude.

Pour le suivi des patients seront conservés : le patronyme et les coordonnées téléphoniques et postales de chacun.

Les patients seront également informés à leur demande des résultats globaux de la recherche.

## 7.9 Amendements au protocole de l’étude

Il n’y aura pas d’altération ou de changement à ce protocole sans accord de l’ensemble des investigateurs et du promoteur. En cas d’un tel accord, si les modifications prévues modifient les aspects éthiques ou médico-scientifiques de l’étude (critère d’évaluation, adjonction d’un nouveau centre,....), elles devront faire l’objet d’un amendement qui sera joint au protocole. Tout amendement au protocole de l’étude fera l’objet d’un nouvel avis du CCP et d’une information auprès du CCTIRS et de la CNIL.

## 7.10 Extension de la recherche

Toute extension de l’étude (modification profonde du schéma thérapeutique ou des populations incluses, prolongation des traitements et ou des actes thérapeutiques non prévus initialement dans le protocole) devra être considérée comme une nouvelle recherche.

## 7.11 Archivage des données

Les documents de l’étude (les cahiers d’observation, les feuilles de corrections différées et la correspondance) seront légalement conservés par l’investigateur pendant une période minimale de 15 ans.

Seront également archivés les documents suivants :

- pré requis

- protocole avec ses annexes et amendements

- cahiers d’observation avec documents annexes et fiches de correction différées

- documents de suivi de l’étude clinique

- toute pièce administrative liée à l’étude

- rapport d’analyse statistique

- rapport d’étude.

Le promoteur gardera un duplicata de ces informations pendant 15 ans également.

## 7.12 Rapport final de la recherche

Le rapport final de l’étude sera écrit en collaboration par l’investigateur et le biostatisticien pour cette recherche. Ce rapport sera soumis à chacun des collaborateurs pour avis. Une fois qu'un consensus aura été obtenu, la version finale devra être avalisée par la signature de l’investigateur et adressée au promoteur dans les meilleurs délais après la fin effective de l’étude.

## 7.13 Budget de l’étude

En l’absence de financement institutionnel une subvention des Laboratoires Sanofi-Aventis de 10 000 Euros pour la conception, l’impression des livrets et la réalisation de l’étude a été obtenue.

## 7.14 Publications et propriétés des données

Le promoteur, CHU de Clermont-Ferrand, est propriétaire des données et aucune utilisation ou transmission à un tiers ne peut être effectuée sans son accord préalable. seront premiers signataires des publications, les personnes ayant participé à l’élaboration du protocole, à son déroulement ainsi qu’à la rédaction des résultats. Le promoteur doit être mentionné comme tel et comme soutien financier le cas échéant.

**8- REFERENCES BIBLIOGRAPHIQUES**

[1] Viton JM, Atlani L, Mesure S, Franceschi JP, Massion J, Delarque A, Bardot A. Reorganization of equilibrium and movement control strategies in patients with knee arthritis. Scand J Rehabil Med. 1999 ; 31: 43-8.

[2] Fortin PR, Clarke AE, Joseph L et al. Outcomes of total hip and knee replacement: preoperative functional status predicts outcomes at six months after surgery. Arthritis Rheum. 1999 ; 42 : 1722-8.

[3] Coudeyre E., Jardin C., Givron P., Ribinik P., Revel M., Rannou F. – Could preoperative

rehabilitation modify postoperative outcomes after total hip and knee arthroplasty?

Elaboration of French clinical practice guidelines. Ann. Readapt. Med. Phys., 2007, 50,

189-197.

[4] HAS. – Critères de suivi en rééducation et d’orientation après arthroplastie totale du genou.

Service des recommandations professionnelles, 2008, Paris, France.

[5] Coudeyre E. Enhancing patient education in physical medecine and rehabilitation practice. Ann Phys Rehabil Med. 2009;52(7-8)

[6] Buchbinder R, Jolley D, Wyatt M. Population based intervention to change back

pain beliefs and disability: three part evaluation. BMJ. 2001 Jun

23;322(7301):1516-20.

[7] Mc Clune. Mc Clune T, Burton AK, Main C. Evaluation of an evidence based patient educational booklet for management of whiplash associated disorders.

Emerg Med J 2003;20:1–4.

[8] ANAES. Information des patients. Recommandations aux médecins. Service des recommandations et références professionnelles, 2000, Paris, France

[9] Eschalier B, Descamps S, Boisgard S, Pereira B, Lefevre-Colau MM, Claus D, Coudeyre E. Validation of an educational booklet targeted to patients candidate for total knee arthroplasty. Orthop Traumatol Surg Res. 2013 May;99(3):313-9.
