# Supplementary material for: Randomized blinded trial of standardized written patient information before total knee arthroplasty
Source: PLoS One. 2017 Jul 5;12(7):e0178358. doi: 10.1371/journal.pone.0178358 (PMC5497941; doi:10.1371/journal.pone.0178358)
Supplement: S2 Protocol — (DOC) [file pone.0178358.s004.doc]

**CENTRE HOSPITALIER UNIVERSITAIRE DE CLERMONT-FERRAND**

**CLINICAL TRIAL PROTOCOL**

**Randomized Blinded Trial of Standardized Written Patient Information before Total Knee Arthroplasty**

**EPOP Study**

**Sponsor :** CHU de Clermont-Ferrand

**Principal Investigator :** Pr Stéphane Descamps

Service de chirurgie orthopédique et traumatologique

Hôpital Gabriel Montpied - CHU de Clermont-Ferrand

58 rue Montalembert

63003 Clermont-Ferrand cedex 1

**Study setting :** Service de Chirurgie Orthopédique + Antenne de Médecine Physique et de Réadaptation

Hôpital Gabriel Montpied - CHU de Clermont-Ferrand

58 rue Montalembert

63003 Clermont-Ferrand cedex 1

**TABLE OF CONTENTS**

**Page**

**Synopsis** **3**

**1- GENERAL INFORMATION 4**

**2- STUDY RATIONNALE 7**

2.1- Background 7

2.2- Practice guidelines 8

2.3- Impact of information leaflets 8

**3- TRIAL OBJECTIVE AND PURPOSE 8**

3.1-Primary objective 8

3.2- Secondary objectives 8

**4- TRIAL DESIGN 8**

4.1- Leaflet elaboration 9

4.2- Randomized controlled study 10

4.2.1.-Expérimental Plan

4.2.2- Subjects selection

4.2.3- Randomisation

4.2.4- Outcomes

4.3- Design of trial to be conducted 13

4.3.1- For the patients

4.3.2- Bias reduction measures

4.3.3 – Dropped out

**5 STATISTICS 15**

5.1- Subjects number 15

5.2- Data Analysis 15

**6- STUDY FEASIBILITY AND EXPECTED RESULTS 16**

6.1- Study feasibility 17

6.2- Expected results 17

**7 ETHICS AND LEGAL FACTS 18**

7.1- Usual care 17

**7.2- Registration 18**

[**7.3-**](#__RefHeading___Toc157336626) **Source documents 18**

7.4- Quality insurance 18

7.5 Quality management 18

[**7.6-**](#__RefHeading___Toc157336627) **Patients information 19**

[**7.7- CRF**](#__RefHeading___Toc157336628) **report 19**

**7.8- Confidentiality 19**

[**7.9-**](#__RefHeading___Toc157336631) **Protocol modifications 20**

7.10- Trial extension 20

7.11- Archiving 20

[**7.12- Final report**](#__RefHeading___Toc157336632) **21**

7.13- Financing 21

[**7.14- Publications and**](#__RefHeading___Toc157336633) **date properties 21**

**8- REFERENCES 22**

**SYNOPSIS**

| **Title** | Randomized Blinded Trial of Standardized Written Patient Information before Total Knee Arthroplasty. |
| --- | --- |
| **Primary Investigator** | Pr Stéphane Descamps |
| **Investigation Center** | CHU Clermont-Ferrand |
| **Objectives** | **Primary objective**  To evaluate the impact of an information booklet on TKR-related knowledge among patients scheduled for TKR. |
| **Expérimental plan** | Randomized controlled study |
| **Patients number** | 22 patients for intervention group and 22 patients for control group (usual-care verbal informations delivered by the surgeon)  For type I and type II errors of 5% and 10%, respectively, 22 patients are needed per group to detect a difference of at least 2 points on the knowledge score, given that a previous pilot study done by our group showed a standard deviation of 2. |
| **Patient follow up** | From 2 to 3 months depending time between inclusion visit and surgery |
| **Study Duration** | 6 months |
| **Study flow** | - V0 inclusion - Intervention (information leaflet) - Pre-surgical visit - Phone follow-up 6 weeks after surgery |
| **Inclusion and non inclusion criteria** | **Inclusion criteria** :   - age 55 to 75 years, - incapacitating knee osteoarthritis, - scheduled TKA, - ability to understand and cooperate with the study protocol, and informed consent to study participation.   **Non inclusion criteria** :   - institutionalization; - cognitive impairments or behavioral disorders; - difficulties with the French language that would preclude completion of the study assessments; - previous TKA on the same knee; - chronic inflammatory joint disease; and complex TKA. |
| **Outcomes** | **Primary outcome** :  The primary outcome measure was patient knowledge about TKA assessed on the 10-items questionnaire  **Secondary outcomes** :  patient beliefs, surgery-ward stay length, proportion of patients discharged home and patient satisfaction with the information received about each of four items (hospital stay, surgery and surgical risks, available help in terms of money or home assistance, and ability to make changes to the home environment), overall patient satisfaction with the information received. |

Kee words : knee osteoarthritis, total knee replacement, patient therapeutic education, information booklet, pre and post surgery rehabilitation.

**1- GENERAL INFORMATIONS**

**RESEARCH TITLE** : Randomized Blinded Trial of Standardized Written Patient Information before Total Knee Arthroplasty. (EPOP study).

**PROMOTION**

CHU de Clermont-Ferrand

**Délégation à la Recherche Clinique & à l’Innovation**

**Direction Générale Adjointe**

**Villa annexe IFSI**

**58 rue Montalembert**

**63003 Clermont-Ferrand Cedex 1**

Tél : 04.73.751.195 / Fax : 04.73.754.730

**PRIMARY INVESTIGATOR**

Pr Stéphane Descamps

Service de Chirurgie Orthopédique et Traumatologique

Hôpital GABRIEL MONTPIED - CHU de Clermont-Ferrand

58 rue Montalembert

63003 Clermont-Ferrand cedex 1

**CO-INVESTIGATORS**

Pr Stéphane Boisgard

Service de Chirurgie Orthopédique et Traumatologique

Hôpital Gabriel Montpied - CHU de Clermont-Ferrand

58 rue Montalembert

63003 Clermont-Ferrand cedex 1

Pr Emmanuel Coudeyre

Service de Médecine Physique et Réadaptation

CHU Clermont-Ferrand, Hôpital Nord

61 route de Chateaugay, BP 30056

63118 Cébazat

Dr Delphine Claus

Service de Médecine Physique et Réadaptation

CHU Clermont-Ferrand, Hôpital Nord

61 route de Chateaugay, BP 30056

63118 Cébazat

**PARTNERS**

Bénédicte Eschalier (Resident)

Physician in charge of the study

Service de Médecine Physique et Réadaptation

CHU Clermont-Ferrand Hôpital Nord

61 rue de Chateaugay, BP 30056

63118 Cébazat

Guillaume Girard (Resident)

Physician in charge of the assessment

Service de Chirurgie Orthopédique et Traumatologique

Hôpital Gabriel Montpied - CHU de Clermont-Ferrand

58 rue Montalembert

63003 Clermont-Ferrand cedex 1

Bruno Pereira, Biostatistician

Direction de la Recherche Clinique

Hôpital Gabriel Montpied - CHU de Clermont-Ferrand

58 rue Montalembert

63003 Clermont-Ferrand cedex 1

**STUDY LOCATION**

Service de chirurgie orthopédique + Antenne de Médecine Physique et Réadaptation

Hôpital Gabriel Montpied - CHU de Clermont-Ferrand

58 rue Montalembert

63003 Clermont-Ferrand cedex 1

**DATA ANALYSIS**

Service de chirurgie orthopédique du CHU de Clermont-Ferrand in collaboration with DRCI (Direction de la Recherche Clinique).

**STUDY PLANNING**

Submission to technical comittee september 2010

Submission to ethical comittee october 2010

Inclusion time: 6 months (november 2010 to may 2011)

Participation duration for each patient: 1 hour (inclusion visit) + 1 hour (follow-up questionnaires)

End of the study : august 2011

Data analysis and final report 2012

# 2- STUDY RATIONNALE

## 2.1 Background

## Knee osteoarthritis results in loss of function due to muscle weakness, mobility and balance impairments, and cardiorespiratory deconditioning [1]. Knee osteoarthritis is the main reason for total knee arthroplasty (TKA), and the number of TKA procedures for knee osteoarthritis is expected to increase by 70% between 2005 and 2030 in the US. TKA improves both function and pain. Pain and functional status before TKA predict the quality of the postoperative recovery [2].

## Pré-operative rehabilitation could improve patient preparation, post-operative function and disability and also contribute to hospital length of stay reduction [3].

2.2 Practice guidelines

Preoperative rehabilitation program, comprising at least physical therapy and education, is recommended by the “Société Française de Médecine Physique et Réadaptation” (SOFMER) before total hip and knee arthroplasty [3]. Occupational therapy could be combined with patient home visits. Isolated physical therapy before total knee arthroplasty (TKA) is not recommended. Multidisciplinary rehabilitation comprising at least occupational therapy and education is desirable for the most fragile patients because of major disability, co-morbidity or social problems.

Pre-operative rehabilitation could associate patient therapeutic education, analytic work and respiratory physiotherapy [4].

Content of informations to be delivered to patients are most often unprecised and not standardized.

2.3 Interest to use a standardized booklet

Printed materials are optimal for disseminating consensual information as a means of improving patient information [5,6]. Many patient information documents are available. However, most of them were developed by the industry, with no prior multidisciplinary discussions and no reference to established evidence or recommendations by learned societies. To the best of our knowledge, there is no widely available information document in French designed for patients awaiting TKA and having a validated content. This aim of patient therapeutic education is to modify patients’ knowledge and beliefs. We therefore have developed an educational booklet according to the method described by McClune et al. [7] and in compliance with guidelines issued by the French National Authority for Health [8] about drafting patient information documents. Development of the booklet took place in five phases and the impact of the booklet have been assessed in an open study [9]

**3- STUDY OBJECTIVE**

The primary purpose of our study is to evaluate the impact of an information booklet on TKA-related knowledge among patients scheduled for TKA.

The secondary purposes are to measure the effect of the booklet on patient beliefs, surgery-ward stay length, discharge-to-home rate, and patient satisfaction.

**4- EXPERIMENTAL PLAN**

Monocentric prospective randomized controlled study, uscual-care information delivered by the orthopaedic surgeon + an information leaflet.

4.1 Intervention

The leaflet was written in accordance with seven steps Mc Clune methodology [7] and Haute Autorité de Santé (HAS) Guidelines [8] with litterature review completed by multidisciplinary experts advice. It has been previously published [9].

4.2 Randomized controlled study

4.2.1 Experimental plan and justification

We will implement a single-center randomized controlled trial comparing:

1) usual care provided by the surgeon in charge of the patient

2) usual care lavished by provided by the surgeon in charge of the patient plus a single educational support in the form of an information booklet.

The randomized controlled trial is the gold standard for therapeutic evaluation. Patients randomized to the control group will not have a specific treatment outside usual information provided by their surgeon. This strategy will represent the most often situation in routine clinical practice and take into account the lack of standard validated information.

By the way, it won’t be possible to blind patients, surgeons, and nurses in charge of patients. Therefore, there will be a risk of bias performance and evaluation. To account for these risks, we will arrange an independent evaluation by a physician not involved in the management of patients.

Patients will be blinded about the hypothesis. They will be informed that the objective of this study will be to compare different pre-operative strategies. However, the content of each take charge and the hypothesis of the study they will not be detailed. This strategy should limit bias, particularly the risk of bias assessment. This method is ethically acceptable because patients will be informed that for scientific reasons we can not explain all the hypotheses of the study. Patients will all be informed at the end of the study hypotheses and results of the study if they wish.

4.2.2 Subjects selection

Patients will be recruited at the Orthopaedic Surgery and Trauma Department of the teaching hospital in Clermont-Ferrand, France, among the total population of patients scheduled for TKA

Inclusion criteria will be :

- age 55 to 75 years,
- incapacitating knee osteoarthritis, s
- cheduled TKA,
- ability to understand and cooperate with the study protocol, and informed consent to study participation.

Non inclusion criteria will be :

- institutionalization;
- cognitive impairments or behavioral disorders;
- difficulties with the French language that would preclude completion of the study assessments;
- previous TKA on the same knee;
- chronic inflammatory joint disease; and
- complex TKA.

## 4.2.3 Outcomes

a)- Main outcome

The primary outcome measure will be patient knowledge about TKA assessed on the 10-item questionnaire used in our earlier study [9]. This criteria will be assessed at inclusion, just before surgery and 6 weeks after surgery

b)- Secondary outcomes

Secondary outcome measures will include patient beliefs about topics addressed in the booklet, assessed using a 4-level Likert scale. Surgery-ward stay length, the proportion of patients discharged home and patient satisfaction with the information received about each of four items (hospital stay, surgery and surgical risks, available help in terms of money or home assistance, and ability to make changes to the home environment) assessed using a 4-level Likert scale will also be assessed. In addition, overall patient satisfaction with the information received will be assessed

## 4.3 Design of trial to be conducted

4.3.1 For the patients

***Inclusion visit***

Screening and inclusion will be made by the physician in charge of the study. After surgeon agreement, the physician in charge of the study will met every patients scheduled for TKR ; This step will permit to check inclusion criteria and propose patients to be enroled in the study.

If the patient is interested to take part of the study, and after a one month period of reflection (time between surgical consultation programming intervention and preoperative anesthesia consultation), a new visit will be organized at the time of anesthesia consultation. During this consultation, the eligibility criteria will be checked, the patient will be informed, and after obtaining its consent will be randomized.

The following data will be collected in the case report:

- Demographic data (age, gender)

- Socio-professional data (socioeconomic background, occupation and employment status (active, off work, invalidity))

- Functional Index: WOMAC scale

***Assessments****:*

Assessments will be conducted using self-reported questionnaires after the inclusion visit, the day before surgery and 6 weeks postoperatively by a telephone interview. The physician in charge of the study will handle the initial note and the patient number on the CRF. To minimize bias, especially for interpretation and data collection, the assessment visits will be carried out by the assessment physician.

## 4.3.2 Bias reduction measures

Randomization :

An individual randomization by subject will be realized. A strata randomization list will be based on age, gender and history of TKR. The numbering will be made chronologically. Randomization will be performed after the inclusion via the computerized medical record managed under I2000 (Patient Management Software Clermont-Ferrand Hospital).

Single blind :

This study will be conducted single-blind regarding the procedure, because the patient receiving an information booklet can not ignore it. The physician in charge of the assessment will not know which group the patient was included.

Contamination bias will be negligible, randomization occuring at least one month prior to surgery, at the end of the consultation of anesthesia. The booklet will be given to the patient just after the consultation of anesthesia. Furthermore, patient assessment will be performed upon admission into the orthopaedic ward limiting interaction between patients and physicians and nurses of surgery.

## 4.3.4 Dropped out

Every patient will be free to refuse to participate in the study without consequences for its management.

A patient released prematurely from the study in the following cases:

- The person decides not to participate in the study.

- The person is not compliant to perform the evaluations planned in the protocol.

For early dropped out from the study, date and reason will be indicated in the CRF of the subject specification. Each retiring prematurely subject of the study should be replaced by the inclusion of a new person.

# 5- STATISTICS

5.1 Subjects number

For type I and type II errors of 5% and 10%, respectively, 22 patients were needed per group to detect a difference of at least 2 points on the knowledge score, given that a previous pilot study done by our group showed a standard deviation of 2.

5.2 Data analysis

Statistical analyses will be performed using Stata software, version 12 (StataCorp, College Station, TX, USA). An intention to treat analysis will be made. The tests will be two-sided, with α=0.05. Patient characteristics will be described in each group as mean±SD or median (interquartile range, IQR) group for continuous variables, depending on distribution, and as the number of patients (%) for categorical variables.

Groups will be compared using the Chi-squared or Fisher exact test for categorical variables and Student t-test or Mann-Whitney test for quantitative variables. Distribution will be assessed using the Shapiro-Wilk test and homoscedasticity using the Fisher-Snedecor test.

To study changes in knowledge scores over time, we will build a mixed model taking into account within- and between-patient variability, considering the patient as a random-effect (slope and intercept) and to study randomization group, time and their interaction as fixed effects considering an adjustment on physical activity. Paired variables within groups will be evaluated using the paired t-test or Wilcoxon test for quantitative parameters and the Stuart-Maxwell test for categorical variables.

**6- STUDY FEASIBILITY AND EXPECTED RESULTS**

6.1 Study feasability

Investigators involved in the project have extensive expertise in the management of patients with knee osteoarthritis, and are competent in the educational approach for total knee replacement patients.

The number of subjects was set at 22 subjects per group. This recruitment will be among a cohort of 200 patients operated over a year at the University Hospital of Clermont-Ferrand.

6.2 Expected results

We expect a significant improvement in patient knowledge and by the way a reduction of their beliefs and an improvement of their satisfaction with the information received.

**7- ETHICS AND LEGAL FACTS**

## 7.1 Usual care

The techniques and methods used in this research are usually conducted, they can be within the scope of research to evaluate routine care as defined by Act No. 2004-806 of 9 August 2004 (Article L1121-1, 2nd paragraph and section R1121-3 the code of public health/ loi n°2004-806 du 9 août 2004 (article L1121-1, 2° alinéa et article R1121-3 du code de la santé publique).

## 7.2 Registration

## Comité de Protection des Personnes

The protocol and the Subject informed form and consent will be submitted to The Independent ethics committee (CPP Sud-Est VI) and written approval from the Chair of the Ethics Committee is required before the initiation of the study.

**CCTIRS et CNIL**

The notification of the approval will be forwarded to the French authority « Traitement de l’Information en matière de Recherche dans le domaine de la Santé » (CCTIRS) and to «  Comission Nationale Informatique et Libertés (CNIL) ». A request for authorization will be sent by the sponsor to CNIL and CCTIRS before the start of the study.

Patients won’t received any grants to take part to the study.

The study will be conducted in accordance to good clinical practices and will respect the principles of Helskinki Declaration (Tokyo 2004, revised).

## 7.3 Source documents

Before starting the study, the investigator shall provide the representative of the sponsor of the research a copy of his curriculum vitae dated and signed with registration number of the College of Physicians.

The version of the protocol, annexes will be jointly signed by the investigator and the sponsor representative. If applicable, the scientific manager is also a signatory.

With each new version of the protocol, made necessary by amendments and / or requests of the authorities, a new number and date will be assigned and the same signatures collected.

Each investigator will undertake to fulfill the obligations of the law and conduct research according to Good Clinical Practice.

## 7.4 Quality insurance

The Clinical research assistant commissioned by the sponsor is responsible for inspection of the case report form at regular intervals, according to the monitoring plan of the study, throughout the study to ensure adherence to the protocol, compliance with the source documents, data consistency, and adherence to regulations on the conduct of clinical research.

The Clinical research assistant commissioned must have access to subject’s medical file and other records related to the study required to verify the case report forms of the study.

## 7.5 Quality control

The investigator is responsible for the authenticity of collected data as part of the study and accepts the legal provisions allowing the sponsor of the study to develop a quality control.

The investigator and coordinator agree to make themselves available for the monitoring visits. During this visits, the following documents will be reviewed:

- Informed consent

- Compliance with the protocol and procedures defined therein

- Quality of collected data in the case report forms : accuracy, missing data, data consistency with the source documents

- Product management

## 7.6 Patients information

Subjects will be informed fully and fairly, in understandable terms, about the objectives, the constraints of the study, the potentials risks involved, and monitoring measures, security, their rights to refuse to participate in the study and the possibility to withdraw at any time.

All these informations must be listed on the informed consent given to the subjects.

Subjects may at any time exercise the right to access, rectification and opposition to the transmission of electronic data for from the responsible of the study. This right can be exercised directly or through a physician of their choice concerning their medical data ( Law of 4 March 2002 on patients' rights and quality of the health system / loi du 4 mars 2002 relative aux droits des malades et à la qualité du système de santé).

## 7.7 CRF report

CRFs are records of data on each subject as defined by the study protocol. Entries on CRFs shall be made complete, legible and correct using a ball-point pen. Any mistakes shall be corrected by drawing a line over the old entry and by initialling and dating next to the correction. The last page of each visit shall be signed and dated by the investigator to indicate the overall responsibility.

## 7.8 Confidentiality

In accordance with GCP and with the national data protection laws, all information concerning the subjects in the study must be treated as strictly confidential by all persons involved in the study including the clinical, medical and statistical monitor.

Data confidentiality is ensured by the use of the first three letters of the name and the first three letters of the first name of the patient on all documents necessary for research associated with a patient number (given in order of inclusion) or deletion by appropriate means. A list of correspondence between the registered members and the patient number will be established and kept by the physician in charge of the study. It will be destroyed at the end of the study.

Patients will also be informed at their request of the overall research results

## .7.9 Protocol modification

There will be no alterations or changes to the protocol without agreement of all investigators and sponsor.

If such an agreement, the planned changes will constitute an amendment that will be attached to the protocol.

Any amendment must be notified to the ethic committee if the planned changes affect the ethical or medical-scientific study (evaluation criteria, addition of a new center ….). Minor modifications do not require a review of the ethic committee and CCTIRS and CNIL will be informed.

## 7.10 Trial extension

Any extension of the study (deep modification of inclusion criteria, prolongation of treatment and or unexpected therapeutic procedures) will be considered a new trial.

## 7.11 Archiving

The following documents will be archived by the name of the study in the CHU of Clermont-Ferrand to the end of the period of practical use (15 years).

These documents are:

- Protocol and annexes, any amendments,

- Information and consent forms signed (originals)

- Individual data (authenticated copies of raw data)

- Follow-up documents

- Statistical analysis

- Final report of the study

At the end of the period of practical use, all documents to be archived, as defined in the procedure PG.06.005 “Managing the documentation of protocols” of the University Hospital of Clermont-Ferrand will be transferred to central archives and will be under the responsibility of the Hospital for 15 years after the end of the study according to institutional practices.

No destruction can be performed without the consent of the sponsor; At the end of the 15 years, the sponsor will be consulted for destruction. All data, all documents and reports may be subject to audit or inspection.

## 7.12 Final report

The final report of the study will be co-written by the investigator and the biostatistician. This report will be submitted to each co-investigators for advice. Once a consensus has been obtained, the final version must be endorsed by the signature of the investigator and the sponsor addressed as soon as possible after the effective end of the study.

## 7.13 Financing

A grant from Sanofi-Aventis Laboratories (10 000 Euros) had been obtained for the design, printing booklets and conduct of the study .

## 7.14 Publication and data properties

Investigator will have full and unrestricted access to the database with all anonymized data. It is intended to publish the results of the clinical trial collectively (no individual report or publication will be allowed).

Neither the Investigator nor his agents, consultants, associates or employees shall, directly or indirectly, originate, issue or disclose news releases or any type of announcements, whether written or oral, or organize any presentation or issue any publications regarding the study, and/or any information, data, results, inventions or discoveries made or obtained during the study, without the prior written consent of sponsor, which consent shall not be unreasonably withheld.

**8- REFERENCES**

[1] Viton JM, Atlani L, Mesure S, Franceschi JP, Massion J, Delarque A, Bardot A. Reorganization of equilibrium and movement control strategies in patients with knee arthritis. Scand J Rehabil Med. 1999 ; 31: 43-8.

[2] Fortin PR, Clarke AE, Joseph L et al. Outcomes of total hip and knee replacement: preoperative functional status predicts outcomes at six months after surgery. Arthritis Rheum. 1999 ; 42 : 1722-8.

[3] Coudeyre E., Jardin C., Givron P., Ribinik P., Revel M., Rannou F. – Could preoperative

rehabilitation modify postoperative outcomes after total hip and knee arthroplasty?

Elaboration of French clinical practice guidelines. Ann. Readapt. Med. Phys., 2007, 50,

189-197.

[4] HAS. – Critères de suivi en rééducation et d’orientation après arthroplastie totale du genou.

Service des recommandations professionnelles, 2008, Paris, France.

[5] Coudeyre E. Enhancing patient education in physical medecine and rehabilitation practice. Ann Phys Rehabil Med. 2009;52(7-8)

[6] Buchbinder R, Jolley D, Wyatt M. Population based intervention to change back

pain beliefs and disability: three part evaluation. BMJ. 2001 Jun

23;322(7301):1516-20.

[7] Mc Clune. Mc Clune T, Burton AK, Main C. Evaluation of an evidence based patient educational booklet for management of whiplash associated disorders.

Emerg Med J 2003;20:1–4.

[8] ANAES. Information des patients. Recommandations aux médecins. Service des recommandations et références professionnelles, 2000, Paris, France

[9] Eschalier B, Descamps S, Boisgard S, Pereira B, Lefevre-Colau MM, Claus D, Coudeyre E. Validation of an educational booklet targeted to patients candidate for total knee arthroplasty. Orthop Traumatol Surg Res. 2013 May;99(3):313-9.
